# Population genetics and forensic utility of 23 autosomal PowerPlex Fusion 6C STR loci in the Kuwaiti population

**DOI:** 10.1038/s41598-021-81425-y

**Published:** 2021-01-21

**Authors:** Mahdi Haidar, Fatimah A. Abbas, Hussain Alsaleh, Penelope R. Haddrill

**Affiliations:** 1 Centre for Forensic Science, Department of Pure and Applied Chemistry, University of Strathclyde, 204 George Street, Glasgow, G1 1XW Scotland, UK; 2 Kuwait Identification DNA Laboratory (KIDL), General Department of Criminal Evidence, Ministry of Interior, Kuwait City, Kuwait

**Keywords:** Genetic markers, Genotype, Population genetics

## Abstract

This study evaluates the forensic utility of 23 autosomal short tandem repeat markers in 400 samples from the Kuwaiti population, of which four markers (D10S1248, D22S1045, D2S441 and SE33) are reported for the first time for Kuwait. All the markers were shown to exhibit no deviation from Hardy–Weinberg equilibrium, nor any linkage disequilibrium between and within loci, indicating that these loci are inherited independently, and their allele frequencies can be used to estimate match probabilities in the Kuwaiti population. The low combined match probability of 7.37 × 10^–30^ and the high paternity indices generated by these loci demonstrate the usefulness of the PowerPlex Fusion 6C kit for human identification in this population, as well as to strengthen the power of paternity testing. Off-ladder alleles were seen at several loci, and these were identified by examining their underlying nucleotide sequences. Principal component analysis (PCA) and *STRUCTURE* showed no genetic structure within the Kuwaiti population. However, PCA revealed a correlation between geographic and genetic distance. Finally, phylogenetic trees demonstrated a close relationship between Kuwaitis and Middle Easterners at a global level, and a recent common ancestry for Kuwait with its northern neighbours of Iraq and Iran, at a regional level.

## Introduction

The State of Kuwait is located on the Arabian Gulf in the northwest of the Asian continent and in the heart of the Middle East. Kuwait is bordered by the Kingdom of Saudi Arabia in the south, the Republic of Iraq in the north and west, and Iran in the east, across the Persian Gulf Sea. Kuwait’s population is about 4.8 million, which includes 1.4 million Kuwaiti nationals and 3.4 million foreign nationals, according to the 2019 census (https://www.paci.gov.kw/stat/Default.aspx). Currently, forensic DNA analysis in Kuwait is carried out by the Kuwaiti Identification DNA Laboratory (KIDL) using only short tandem repeat (STR) markers, including autosomal STRs, Y-chromosome STRs (Y-STRs) and X-chromosome STRs (X-STRs). Autosomal STRs are routinely used both for identification of individuals and paternity testing, whereas Y-STRs and X-STRs are used less frequently, and only for specific scenarios.

To date, few papers have been published investigating the forensic utility and genetic diversity of autosomal STR markers in the Kuwaiti population. In 2008, Alenizi and colleagues reported the allele frequencies of 15 STR loci included in the AmpFℓSTR Identifiler kit (Thermo Fisher Scientific, MA, USA)^1^. Based on these 15 STRs, the F_ST_ distances between Kuwaiti nationals and foreign nationals from seven other populations residing in Kuwait were found to be consistent with their geographical distances^2^. Another recent study investigated the forensic utility of 25 autosomal STRs included in two separate kits: the PowerPlex CS7 system and the PowerPlex 21 system (Promega Corporation, WI, USA)^3^. Although these existing STRs are efficient for analysing cases of simple relationships, more STRs are increasingly required, particularly for complex paternity cases or to increase the discrimination power in cases of partial DNA profiles and DNA mixtures.

Recently, Promega launched the PowerPlex Fusion 6C kit, a six-dye kit that can amplify 27 loci, including the 20 autosomal loci in the expanded CODIS set (CSF1PO, FGA, TH01, TPOX, vWA, D1S1656, D2S1338, D2S441, D3S1358, D5S818, D7S820, D8S1179, D10S1248, D12S391, D13S317, D16S539, D18S51, D19S433, D21S11, and D22S1045)^4^, three additional autosomal STRs (PentaE, PentaD, and SE33) to increase the power of discrimination, two sex chromosome markers (Amelogenin and DYS391), and two rapidly mutating Y-STRs (DYS570 and DYS576)^5^. The PowerPlex Fusion 6C kit was validated by multi-laboratory evaluation following SWGDAM guidelines^5^.

Before utilising this kit for criminal and relationship cases in Kuwait, population and forensic statistical data for the loci in the kit must be evaluated. In this study, we aim to increase the amount of genetic data available for the Kuwaiti population, using the 23 autosomal STRs in the PowerPlex Fusion 6C kit, of which four loci (D10S1248, D22S1045, D2S441 and SE33) have not been reported before for Kuwait. In addition, we aim to evaluate the forensic utility of these autosomal STRs in this underrepresented region, and to investigate the utility of these markers in population genetic differentiation by examining the genetic distance between the Kuwaiti population and other global populations for which data are available.

## Materials and methods

### Samples and genotyping

Blood samples were collected on Whatman FTA cards (GE Healthcare Life Sciences, IL, USA) from 400 unrelated Kuwaiti (253 males and 147 females). DNA was amplified directly, without quantification, from a 1.2 mm FTA card punch, according to the directions in the PowerPlex Fusion 6C manual, using a SureCycler 8800 thermal cycler (Agilent Technologies, CA, USA). Detection and separation of the DNA fragments were carried out using an Applied Biosystems 3500 Genetic Analyzer (Thermo Fisher Scientific) with the internal lane standard WEN ILS 500 and allelic ladder provided with the PowerPlex Fusion 6C kit. Genotype determination and allele calling for only the 23 autosomal loci were carried out using *GeneMapper ID-X* software version 1.4 (Thermo Fisher Scientific).

### Statistical analysis

Data analysis was carried out for the 23 autosomal loci only (the sex chromosomes are not included in this paper). *Arlequin* statistical software version 3.5 was used to calculate allele frequencies, to test for linkage disequilibrium, and to test for deviation from the Hardy–Weinberg Equilibrium^6^. Forensic parameters, including the random match probability (RMP), discrimination power (DP), power of exclusion (PE), typical paternity index (TPI) and polymorphic information content (PIC), were calculated using *STRAF* (http://cmpg.unibe.ch/shiny/STRAF/), an online tool for STR data analysis^7^.

### Intra-population genetic structure among Kuwaitis

Countries in the Arabian Peninsula, including Kuwait, have a high rate of consanguineous marriage, which causes differential distribution of alleles among families and tribes, resulting in population genetic stratification^8, 9^. Newly presented markers therefore must be assessed for the presence of any population structure, to avoid calculation of forensic parameters using inaccurate allele frequencies taken from the total population, rather than the relevant subpopulation. Stratification also negatively impacts discrimination power, because the chance of random individuals possessing similar genotypes is higher within a subpopulation, than within the total population^10^. Two methods were therefore used to detect genetic structure in the population, principal component analysis (PCA), and a Bayesian-based method implemented in *STRUCTURE* version 2.3.4^11–13^.

In order to demonstrate whether these two methods were able to cluster the samples into their real subpopulations, each sample was categorised into one of three ancestral subgroups (*K* = 3) based on the donor’s surname. It has previously been found that the Kuwaiti population is mainly composed of settlers coming from three different regions: the Arabian Peninsula (from Saudi Arabia), the desert (representing nomadic tribes), and Persian countries (mainly from Iran)^9, 14–17^. On this basis, the samples were categorised into three groups: KW-1 (n = 162) representing individuals originating from the Arabian Peninsula, KW-2 (n = 163), which consists of those coming from Persian countries and Iraq (north), and KW-3 (n = 75) composed of Bedouin individuals coming from nomadic tribes. PCA was carried out on allele frequencies at the 23 autosomal STR loci for the different population groups KW-1–KW-3 using R software^33^ and visualised using the *factoextra* package^34^*.*

In contrast to PCA, which is an unsupervised clustering algorithm, *STRUCTURE* (a Bayesian-based approach) takes a range of numbers of populations (*K*) in order to calculate the proportion of the genome of each individual in the sample originating from each inferred population^11^. *STRUCTURE* software calculates the likelihood of the data (X) for range of *K* values, and the true number of *K* is determined by the maximal value of Ln P(X|K). However, it was found by Evanno et al.^18^ that the maximal value does not always provide the correct number of *K* in the data. Instead, the maximal value of the rate of change (Delta *K*) in the Ln P(X|K) between successive *K* values accurately infers the true number of genetic clusters in the data^18^. As such, both Ln P(X|K) and Delta *K* at each *K* were calculated and reported. *STRUCTURE* was run without population information, as recommended in the *STRUCTURE* documentation, in order to check whether the results approximately agreed with the separation of samples into their subgroups. Thus, the predefined groups (KW-1 to KW-3) were only included as a population label rather than as prior information for the analysis. The parameters for the analysis were set as follows: ‘admixture’ and ‘correlated allele frequencies’ models using 100,000 Markov Chain Monte Carlo (MCMC) steps for each run, with the first 100,000 discarded as a burn-in, and the inferred number of *K* was set from 1 to 10. At each *K*, the analysis was repeated five times in order to test the results for consistency. The results were visualised using *CLUMPAK* (Clustering Markov Packager Across K, available at http://clumpak.tau.ac.il/index.html)^19^, and the best *K* was calculated using *STRUCTURE HARVESTER* (available at http://taylor0.biology.ucla.edu/structureHarvester/)^20^.

### Inter-population genetic structure and population relationships

To assess the genetic distance between the Kuwaiti sample and other global populations, PCA was conducted based on allele frequencies of the 23 autosomal loci for 57 global populations grouped into seven continental regions: Africa (AFR), America (AMR), Central and South Asia (C_S_ASIA), the Middle East (ME), Europe (EUR) and East Asia (E_ASIA). Data for the global populations were obtained from the HGDP-CEPH Human Genome Diversity Panel (HGDP-CEPH) using the online forensic STR frequency browser, *popSTR* (http://spsmart.cesga.es/popstr.php)^21–23^. Data from Lebanon (LEB) and an Indian (IND) population from Madhya Pradesh typed for the 23 autosomal loci^24^ were also included in the analysis. Genetic distance was also assessed at a regional level using allele frequencies for the 13 of the 23 autosomal loci (CSF1PO, D13S317, D16S539, D18S51, D21S11, D3S1358, D5S818, D7S820, D8S1179, FGA, TH01, TPOX, vWA) that are shared between the data reported in this study and other studies of Kuwait and neighbouring counties: Kuwait (KW1^3^ and KW2^2^), Iran (IRN^25^ and IRN1^2^), Saudi Arabia (SA^26^ and SA1^27^), Qatar (QAT^28^), Oman (OMN^29^), Yemen (YEM^29^), United Arab Emirates (UAE^30^), Bahrain (BAH^31^), and Iraq (IRQ^32^ and IRQ1^2^). PCA analysis was conducted using R software^33^ and visualised using the *factoextra* package^34^.

In addition to the PCA, we studied the genetic relationship between the Kuwaiti samples and the other populations at both the continental and regional levels, using phylogenetic trees. These trees were constructed using pairwise genetic distances (*D*_*A*_) based on Nei et al.^35^, which were calculated from the allele frequencies of the populations using *POPTREE2* software^36^. The type of phylogenetic trees used were Neighbour-joining (NJ) trees, constructed using *Mega X* software version 10.0.5^37^.

### Ethics statement

The study was performed in accordance with the University of Strathclyde code of practice on investigations involving human beings, and ethical approval (reference number DEC18/PAC06) was granted by the Department of Pure and Applied Chemistry Ethics Committee. Written, informed consent was obtained from all participants prior to sampling.

## Results and discussion

### Allele frequencies and forensic performance

Full PowerPlex Fusion 6C STR profiles were recovered from blood samples taken from 400 Kuwaiti individuals. Table 1 shows the allele frequencies and forensic parameters calculated for these samples. Similar to studies of other global populations^38, 39^, SE33 was the most discriminative locus in the Kuwaiti population, having 45 different alleles (PIC = 0.945). In contrast, TPOX was the least discriminative locus, with only eight different alleles (PIC = 0.616). The calculated combined match probability (CMP) was 7.37 × 10^–30^, meaning that the probability of observing two identical profiles for the 23 autosomal loci in the Kuwaiti population was 1 in 1.36 × 10^29^ The TPI ranged between 1.439 (TPOX) and 8.333 (SE33), and the combined PE was > 99.9999%. These high values indicate the usefulness of the PowerPlex Fusion 6C kit for both human identification and paternity testing in the Kuwaiti population.

**Table 1:** Allele frequencies among 400 Kuwaiti individuals typed at 23 autosomal STR loci in the PowerPlex Fusion 6C kit.

Allele	CSF1PO	D10S1248	D12S391	D13S317	D16S539	D18S51	D19S433	D1S1656	D21S11	D22S1045	D2S1338	D2S441	D3S1358	D5S818	D7S820	D8S1179	FGA	PentaE	PentaD	TH01	TPOX	vWA	SE33
2.2	–	–	–	–	–	–	–	–	–	–	–	–	–	–	–	–	–	–	0.025	–	–	–	–
3.2	–	–	–	–	–	–	–	–	–	–	–	–	–	–	–	–	–	–	0.001	–	–	–	–
5	–	–	–	–	–	–	–	–	–	–	–	–	–	–	–	–	–	0.07	0.001	–	–	–	–
6	–	–	–	–	–	–	–	–	–	–	–	–	–	–	–	–	–	–	0.003	0.279	0.003	–	–
6.3	–	–	–	–	–	–	–	–	–	–	–	–	–	–	–	–	–	–	–	–	–	–	0.001
7	0.001	–	–	–	–	–	–	–	–	–	–	–	–	–	0.02	–	–	0.116	0.009	0.224	–	–	–
8	0.003	–	–	0.119	0.043	–			–	–	–	0.001	–	0.016	0.17	0.009	–	0.041	0.023	0.121	0.51	–	–
9	0.024	0.006	–	0.043	0.168	–	0.001	0.001	–	–	–	–	–	0.063	0.093	0.009	–	0.014	0.214	0.228	0.149	–	–
9.1	–	–	–	–	–	–	–	–	–	–	–	0.001	–	–	0.001	–	–	–	–	–	–	–	–
9.3	–	–	–	–	–	–	–	–	–	–	–	–	–	–	–	–	–	–	–	0.123	–	–	–
10	0.29	–	–	0.059	0.088	0.008	0.001	0.006	–	0.009	–	0.14	–	0.103	0.286	0.078	–	0.059	0.183	0.025	0.098	–	–
10.2	–	–	–	–	–	–	–	–	–	–	–	–	–	–	–	–	–	0.001	–	–	–	–	–
10.3	–	–	–	–	–	–	–	–	–	–	–	–	–	–	–	–	–	–	–	0.001	–	–	–
10.4	–	–	–	–	–	–	–	–	–	–	–	–	–	–	–	–	–	–	0.001	–	–	–	–
11	0.264	0.016	–	0.31	0.313	0.024	0.008	0.098	–	0.159	–	0.395	–	0.279	0.255	0.085	–	0.115	0.19	–	0.218	–	0.003
11.2	–	–	–	–	–	–	–	–	–	0.001	–	–	–	–	–	–	–	–	–	–	–	–	–
11.3	–	–	–	–	–	–	–	–	–	–	–	0.07	–	–		–	–	–	–	–	–	–	–
12	0.348	0.026	–	0.328	0.249	0.115	0.093	0.114	–	0.014	–	0.055	–	0.333	0.151	0.12	–	0.164	0.118	–	0.021	–	0.006
12.2	–	–	–	–	–	–	0.01	–	–	–	–	–	–	–	–	–	–	–	–	–	–	–	0.003
12.3	–	–	–	–	–	–	–	–	–	–	–	0.003	–	–	–	–	–	–	–	–	–	–	–
13	0.061	0.21	–	0.106	0.12	0.17	0.249	0.094	–	0.01	–	0.025	0.005	0.2	0.023	0.253	–	0.1	0.125	–	0.001	0.004	0.023
13.2	–	–	–	–	–	–	0.03	–	–	–	–	–	–	–	–	–	–	–	–	–	–	–	0.001
13.3	–	–	–	–	–	–	–	–	–	–	–	0.003	–	–	–	–	–	–	–	–	–	–	0.001
14	0.01	0.345	–	0.035	0.019	0.144	0.235	0.123	–	0.076	0.004	0.281	0.061	0.008	0.001	0.189	–	0.046	0.069	–	0.001	0.083	0.041
14.2	–	–	–	–	–	–	0.049	–	–	–	–	–	–	–	–	–	–	–	–	–	–	–	–
14.3	–	–	–	–	–	–	–	–	–	–	–	–	–	–	–	–	–	–	–	–	–	–	0.001
14.4	–	–	–	–	–	–	–	–	–	–	–	–	–	–	–	–	–	0.001	–	–	–	–	–
15	–	0.224	0.018	0.001	0.003	0.148	0.126	0.139	–	0.436	0.003	0.021	0.234	–	–	0.191	–	0.071	0.035	–	–	0.123	0.031
15.1	–	–	–	–	–	0.001	–	–	–	–	–	–	–	–	–	–	–	–	–	–	–	–	–
15.2	–	–	–	–	–	0.001	0.084	–	–	–	–	–	–	–	–	–	–	–	–	–	–	–	0.001
15.3	–	–	–	–	–	–	–	0.029	–	–	–	–	–	–	–	–	–	–	–	–	–	–	–
15.4	–	–	–	–	–	–	–	–	–	–	–	–	–	–	–	–	–	0.003	–	–	–	–	–
16	–	0.134	0.015	–	–	0.133	0.061	0.226	–	0.248	0.041	0.005	0.266	–	–	0.05	–	0.048	0.004	–	–	0.245	0.06
16.2	–	–	–	–	–	0.001	0.04	–	–	–	–	–	–	–	–	–	–	–	–	–	–	–	–
16.3	–	–	–	–	–	–	–	0.043	–	–	–	–	–	–	–	–	–	–	–	–	–	–	0.001
16.4	–	–	–	–	–	–	–	–	–	–	–	–	–	–	–	–	–	0.001	–	–	–	–	–
17	–	0.03	0.128	–	–	0.1	0.01	0.058	–	0.043	0.189	–	0.286	–	–	0.015	0.056	–	–	–	0.249	0.073	–
17.2	–	–	–	–	–	0.001	0.004	–	–	–	–	–	–	–	–	–	0.001	–	–	–	–	–	–
17.3	–	–	0.004	–	–	–	–	0.059	–	–	–	–	–	–	–	–	–	–	–	–	–	–	0.003
17.4	–	–	–	–	–	–	–	–	–	–	–	–	–	–	–	–	–	0.001	–	–	–	–	–
18	–	0.008	0.194	–	–	0.085	–	0.001	–	0.005	0.12	–	0.14	–	–	0.003	0.008	0.033	0.001	–	–	0.205	0.089
18.3	–	–	0.019	–	–	–	–	0.009	–	–	–	–	–	–	–	–	–	–	–	–	–	–	–
18.4	–	–	–	–	–	–	–	–	–	–	–	–	–	–	–	–	–	0.001	–	–	–	–	–
19	–	0.001	0.126	–	–	0.046	–	–	–	–	0.119	–	0.006	–	–	–	0.06	0.025	–	–	–	0.081	0.088
19.1	–	–	0.001	–	–	–	–	–	–	–	–	–	–	–	–	–	–	–	–	–	–	–	–
19.2	–	–	–	–	–	–	–	–	–	–	–	–	–	–	–	–	0.003	–	–	–	–	–	0.001
19.3	–	–	0.01	–	–	–	–	0.003	–	–	–	–	–	–	–	–	–	–	–	–	–	–	0.001
20	–	–	0.095	–	–	0.01	–	–	–	–	0.156	–	0.001	–	–	–	0.086	0.025	–	–	–	0.011	0.044
20.2	–	–	–	–	–	–	–	–	–	–	–	–	–	–	–	–	–	–	–	–	–	–	0.003
21	–	–	0.103	–	–	0.008	–	–	–	–	0.048	–	–	–	–	–	0.143	0.005	–	–	–	–	0.016
21.2	–	–	–	–	–	–	–	–	–	–	–	–	–	–	–	–	0.003	–	–	–	–	–	0.02
22	–	–	0.105	–	–	0.003	–	–	–	–	0.05	–	–	–	–	–	0.171	0.003	–	–	–	–	0.008
22.1	–	–	–	–	–	–	–	–	–	–	–	–	–	–	–	–	–	–	–	–	–	–	0.004
22.2	–	–	–	–	–	–	–	–	–	–	–	–	–	–	–	–	0.003	–	–	–	–	–	0.014
23	–	–	0.114	–	–	0.004	–	–	–	–	0.115	–	–	–	–	–	0.179	0.001	–	–	–	–	–
23.2	–	–	–	–	–	–	–	–	–	–	–	–	–	–	–	–	0.003	–	–	–	–	–	0.033
24	–	–	0.039	–	–	–	–	–	–	–	0.085	–	–	–	–	–	0.17	–	–	–	–	–	0.001
24.2	–	–	–	–	–	–	–	–	–	–	–	–	–	–	–	–	0.003	–	–	–	–	–	0.028
25	–	–	0.024	–	–	–	–	–	–	–	0.054	–	–	–	–	–	0.118	–	–	–	–	–	–
25.2	–	–	–	–	–	–	–	–	–	–	–	–	–	–	–	–	0.001	–	–	–	–	–	0.026
26	–	–	0.005	–	–	–	–	–	–	–	0.011	–	–	–	–	–	0.04	–	–	–	–	–	–
26.2	–	–	–	–	–	–	–	–	–	–	–	–	–	–	–	–	–	–	–	–	–	–	0.059
27	–	–	0.003	–	–	–	–	–	0.02	–	0.006	–	–	–	–	–	0.008	–	–	–	–	–	–
27.2	–	–	–	–	–	–	–	–	–	–	–	–	–	–	–	–	–	–	–	–	–	–	0.074
28	–	–	–	–	–	–	–	–	0.144	–	–	–	–	–	–	–	0.001	–	–	–	–	–	–
28.2	–	–	–	–	–	–	–	–	–	–	–	–	–	–	–	–	–	–	–	–	–	–	0.058
29	–	–	–	–	–	–	–	–	0.23	–	–	–	–	–	–	–	0.001	–	–	–	–	–	–
29.2	–	–	–	–	–	–	–	–	0.001	–	–	–	–	–	–	–	–	–	–	–	–	–	0.053
30	–	–	–	–	–	–	–	–	0.224	–	–	–	–	–	–	–	0.001	–	–	–	–	–	–
30.2	–	–	–	–	–	–	–	–	0.019	–	–	–	–	–	–	–	–	–	–	–	–	–	0.053
31	–	–	–	–	–	–	–	–	0.051	–	–	–	–	–	–	–	–	–	–	–	–	–	0.001
31.2	–	–	–	–	–	–	–	–	0.114	–	–	–	–	–	–	–	–	–	–	–	–	–	0.036
32	–	–	–	–	–	–	–	–	0.006	–	–	–	–	–	–	–	–	–	–	–	–	–	–
32.2	–	–	–	–	–	–	–	–	0.119	–	–	–	–	–	–	–	–	–	–	–	–	–	0.019
33	–	–	–	–	–	–	–	–	0.003	–	–	–	–	–	–	–	–	–	–	–	–	–	0.006
33.2	–	–	–	–	–	–	–	–	0.054	–	–	–	–	–	–	–	–	–	–	–	–	–	0.005
34	–	–	–	–	–	–	–	–	0.003	–	–	–	–	–	–	–	–	–	–	–	–	–	0.006
34.2	–	–	–	–	–	–	–	–	0.009	–	–	–	–	–	–	–	–	–	–	–	–	–	0.001
35	–	–	–	–	–	–	–	–	0.001	–	–	–	–	–	–	–	–	–	–	–	–	–	0.003
35.2	–	–	–	–	–	–	–	–	0.003	–	–	–	–	–	–	–	–	–	–	–	–	–	–
36	–	–	–	–	–	–	–	–	0.001	–	–	–	–	–	–	–	–	–	–	–	–	–	0.004
38	–	–	–	–	–	–	–	–	–	–	–	–	–	–	–	–	–	–	–	–	–	–	0.001
Alleles(n)^a^	800	800	800	800	800	800	800	800	800	800	800	800	800	800	800	800	800	800	800	800	800	800	800
Alleles(v)^b^	8	10	17	8	8	18	15	15	17	10	14	12	8	7	9	11	20	24	16	7	8	8	45
Ho^c^	0.725	0.763	0.87	0.753	0.788	0.86	0.823	0.895	0.853	0.705	0.848	0.72	0.78	0.728	0.775	0.835	0.838	0.9	0.838	0.78	0.653	0.808	0.94
He^d^	0.722	0.768	0.885	0.766	0.789	0.879	0.844	0.875	0.844	0.716	0.883	0.737	0.77	0.758	0.793	0.835	0.864	0.912	0.849	0.791	0.661	0.809	0.949
HWE^e^	0.919	0.052	0.599	0.719	0.084	0.108	0.957	0.38	0.478	0.558	0.559	0.578	0.507	0.125	0.136	0.077	0.366	0.359	0.484	0.593	0.339	0.961	0.778
RMP^f^	0.13	0.094	0.026	0.09	0.078	0.029	0.042	0.03	0.046	0.122	0.026	0.109	0.097	0.098	0.076	0.055	0.035	0.016	0.041	0.077	0.161	0.067	0.007
DP^g^	0.87	0.906	0.974	0.91	0.922	0.971	0.958	0.97	0.954	0.878	0.974	0.891	0.903	0.902	0.924	0.945	0.965	0.984	0.959	0.923	0.839	0.933	0.993
PE^h^	0.468	0.531	0.735	0.514	0.576	0.715	0.641	0.785	0.7	0.436	0.69	0.46	0.562	0.472	0.553	0.666	0.67	0.795	0.67	0.562	0.359	0.613	0.878
TPI^i^	1.818	2.105	3.846	2.02	2.353	3.571	2.817	4.762	3.39	1.695	3.279	1.786	2.273	1.835	2.222	3.03	3.077	5	3.077	2.273	1.439	2.597	8.333
PIC^j^	0.67	0.732	0.873	0.731	0.758	0.865	0.825	0.861	0.824	0.675	0.87	0.698	0.731	0.718	0.761	0.813	0.848	0.905	0.83	0.758	0.616	0.78	0.945

^a^Number of alleles surveyed, ^b^number of different allelic variants, ^c^observed heterozygosity, ^d^expected heterozygosity, ^e^Hardy–Weinberg Equilibrium test (*p* value), ^f^random match probability, ^g^discrimination power, ^h^power of exclusion, ^i^typical paternity index, ^j^polymorphic information content.

### Statistical analysis of populations

No significant deviation from the expectations of the Hardy–Weinberg Equilibrium was detected at any locus in the Kuwaiti genotypic data, therefore, the PowerPlex Fusion 6C autosomal STR alleles are independent and can be used to estimate allele frequencies from their genotype frequencies. Association between alleles at all possible pairwise combinations of loci was evaluated using the linkage disequilibrium test. Significant linkage disequilibrium was detected between 22 (of a total of 253) pairs of loci (*p* < 0.05). However, after Bonferroni correction of the significance level using the number of tests (0.05/253 = 0.000198), none of the pairs of loci showed significant linkage disequilibrium, indicating that all loci are statistically independent. Therefore, their allele frequencies can be multiplied together to estimate match probabilities in the Kuwaiti population.

### Off-ladder and novel alleles

Alleles that could not be identified using the *GeneMapper* allelic ladder for the PowerPlex Fusion 6C kit were assigned as off-ladder (OL) alleles, and were observed in 13 samples. These samples were re-amplified for confirmation and all OL alleles were confirmed. OL alleles were observed at the PentaE (5 alleles), PentaD (1 allele), D22S1045 (1 allele), SE33 (5 alleles), and D18S51 (1 allele) loci. The samples were previously sequenced using the Verogen ForenSeq DNA Signature Prep kit (manuscript in preparation), and these data were examined to determine whether the undesignated alleles at the PentaE, PentaD, D22S1045 and SE33 loci could be identified; the repeat structure sequences from this dataset are shown in Table 2 and permitted all alleles to be identified. The D18S51 locus is not included in the ForenSeq kit therefore, its OL allele was identified using the allelic ladder bins created in *GeneMapper* software.

**Table 2:** Off-ladder alleles in the Kuwaiti population identified using sequencing data.

Locus	Repeat Structure	Identified allele
PentaE	AAGA (AAAGA)15	15.4
	(AAAGA)7 AAAA (AAAGA)7	14.4
	AAAGA AA (AAAGA)9	10.2
	AAGA (AAAGA)18	18.4
PentaD	AAAAA AAAG (AAAGA)9	10.4
D22S1045	(ATT)4 AT (ATT)4 ACT (ATT)2	11.2
SE33^a^	CT (CTTT)3 C (CTTT)3 C (CTTT)11 (CTTT)3 CT (CTTT)2	13.3
	CT (CTTT)3 C (CTTT)9T (CTTT)13 CT (CTTT)3 CT (CTTT)2	22.1
	CT (CTTT) C (CTTT)3 C (CTTT)15 (CTTT)2 CT (CTTT)2	17.3

^a^SE33 length allele designation according to Borsuk et al.^41^.

All of the identified alleles have been reported previously in the STRBase database (an online STR database created by the United States National Institute of Standards and Technology (NIST)^40^), except for the PentaD 11.2 allele, which is a novel allele not reported before in the literature.

### Intra-population genetic structure

**Figure 1:**
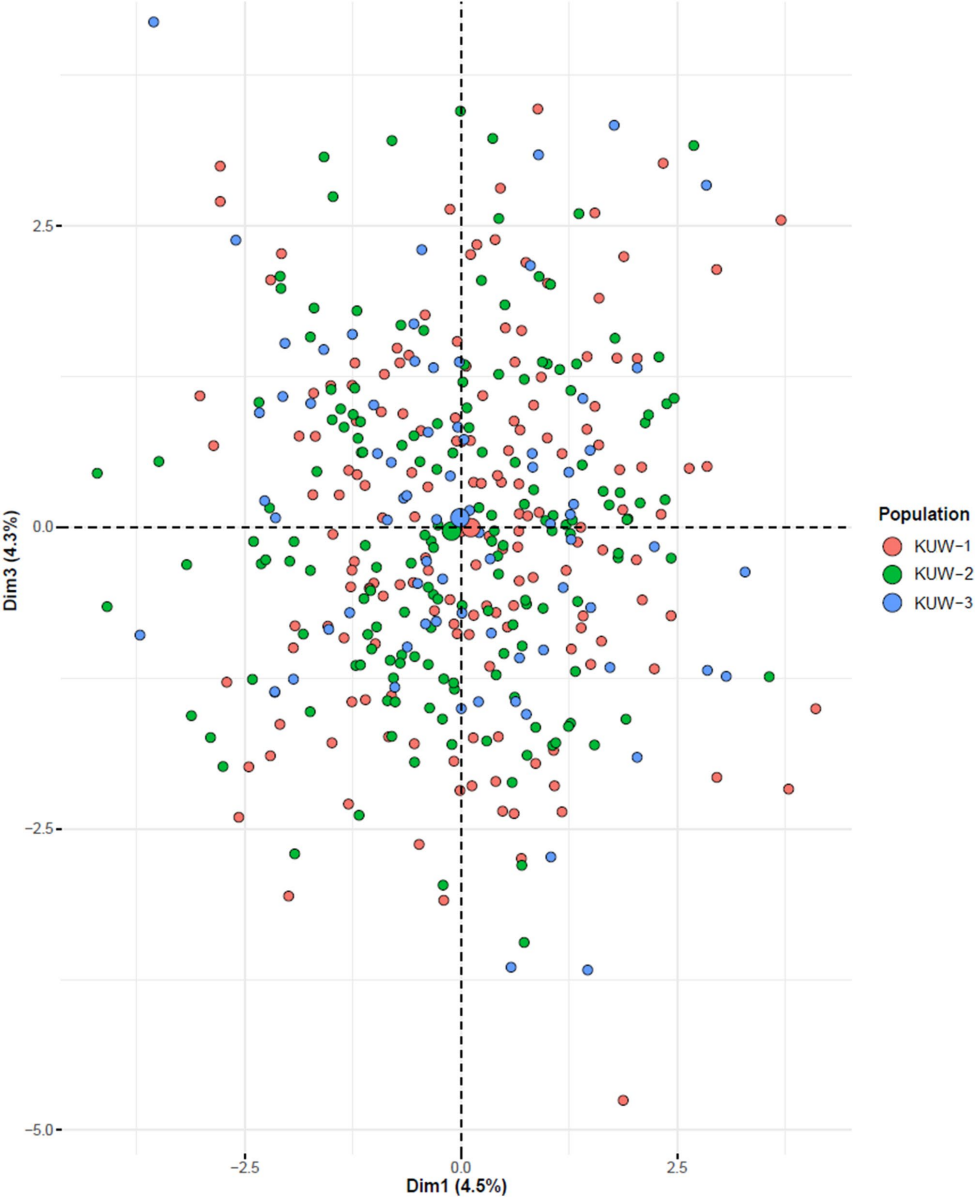
Principal component analysis (PCA) plot showing samples from 400 individuals from the Kuwaiti population, typed for the 23 autosomal STR markers in the PowerPlex Fusion 6C kit. The samples were colour-coded based on their subpopulation of origin KUW-1 to KUW-3. The size of the point represents the number of samples (generated using *factoextra* package^34^ in R software^33^).

Markers that are used for human identification may have weaker discrimination power in populations with genetic structure than in unstructured populations, due to the impact that the presence of subpopulation groups has on the random match probability. This is due to the fact that individuals coming from the same subpopulation groups tend to possess similar alleles, which means the likelihood of seeing random individuals possessing similar genotypes would increase in the presence of genetic structure^10^. Despite the fact that, in this study, no significant deviation from the expectations of the Hardy–Weinberg Equilibrium was detected between the markers, indicating that there is no genetic stratification, it is useful to assess the markers to see if they reveal any genetic clusters within the data. To achieve this, PCA was carried out on the DNA profiles obtained from the Kuwaiti samples for the 23 autosomal PowerPlex Fusion 6C markers. PCA is an unsupervised clustering method that does not require any prior information about the ancestral origin of the samples. Simply, it clusters the samples based on their similarities to each other, forming homogenous clusters of individuals that can be seen on a PCA plot. As expected, the PCA plot (Fig. 1), did not show any pattern of segregation that could be related to the ancestral population of origin of the individuals in the data, indicating that there is no genetic structure within the sample.

Another widely used method to infer population structure in genetic data is the Bayesian-based model implemented in the *STRUCTURE* software, which calculates how likely each individual in the data is to belong to each of a number of *K* (predetermined by the user) populations, and then uses this information to assign individuals into population subgroups^18^. The analysis was run without population information, and the mean log likelihood across five repeated runs of the analysis for each value of *K* (from 2 to 10) was estimated. The results showed inconsistency in estimating the log likelihood at *K* = 5 and over, which is indicated by the high standard deviation (SD), as presented in Supplementary Figure S1A. Based on the method described in Evanno et al.^18^, the most likely inferred value of *K* was 7, as this is the number of populations at which the highest Delta *K* value was recorded (Supplementary Figure S1B).

However, whilst the results indicated that the data is most probable at *K* = 7, there was no clear genetic differentiation between individuals in the sample. This can be seen in Supplementary Figure S2, which shows no clear signal of structuring between the three subpopulation groups, in terms of the proportion of each individual’s genetic ancestry assigned to each population, regardless of the number of populations assumed. This is further supported by the relatively small increases in mean log likelihood and Delta *K* values from *K* = 2 to *K* = 3, suggesting that there is limited evidence for any genetic structuring within the Kuwaiti population sample, in agreement with the PCA analysis above.

### Genetic distance

**Figure 2:**
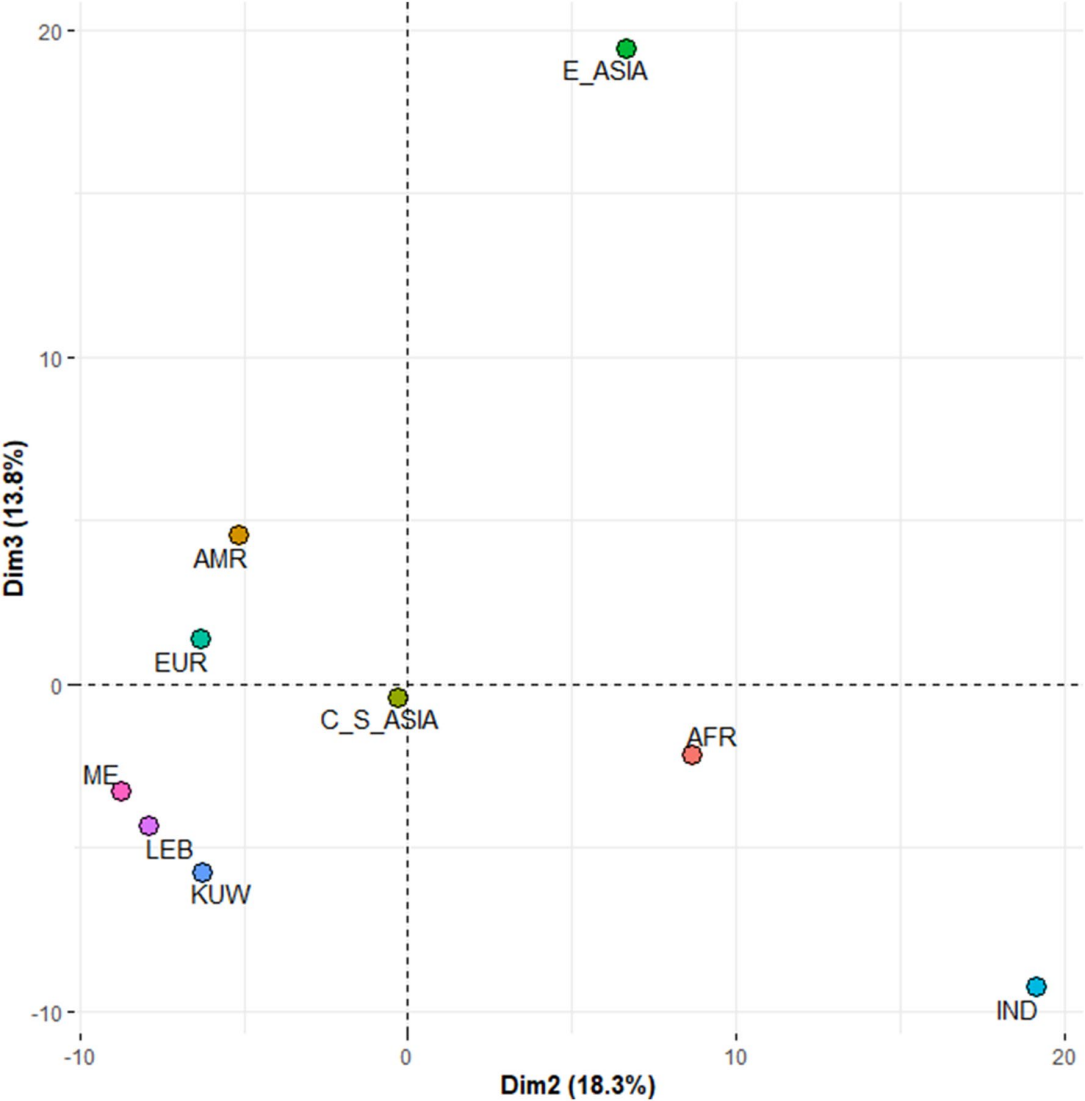
Principal component analysis of genetic distance based on allele frequencies of 23 autosomal PowerPlex Fusion 6C loci shared between the population from Kuwait (KUW; this study) and those from Africa (AFR), America (AMR), Central and South Asia (C_S_ASIA), the Middle East (ME), Europe (EUR), East Asia (E_ASIA), Lebanon (LEB) and India (IND; Madhya Pradesh) (generated using *factoextra* package^34^ in R software^33^).

To investigate the genetic distance between the Kuwaiti population and other global populations, allele frequencies for the 23 autosomal STRs in the PowerPlex Fusion 6C kit were pooled from the HGDP-CEPH global panel, which contains 57 populations grouped into seven global regions, and consists of eight African (N = 507), six American (N = 551), nine Central and South Asian (N = 202), four Middle Eastern (N = 160), 11 European (N = 2135), and 17 East Asian (N = 227) populations. An Indian population (N = 374) and a Lebanese population (N = 505) were also typed for the 23 loci, thus were added to the analysis. Both PCA and phylogenetic analyses were carried out, and the resulting plots characterise the genetic differentiation between populations. The distribution of the populations on the PCA plot (Fig. 2), and the genetic distances between them on the NJ tree (Fig. 4A) show that the Kuwaiti population is genetically closest to the Lebanese and Middle Eastern groups, which includes Mozabite, Druze, Palestinian and Bedouin populations. This is explained by the gene flow between these geographically close locations, which consequently leads to more similar allele frequency distributions among them.

**Figure 3:**
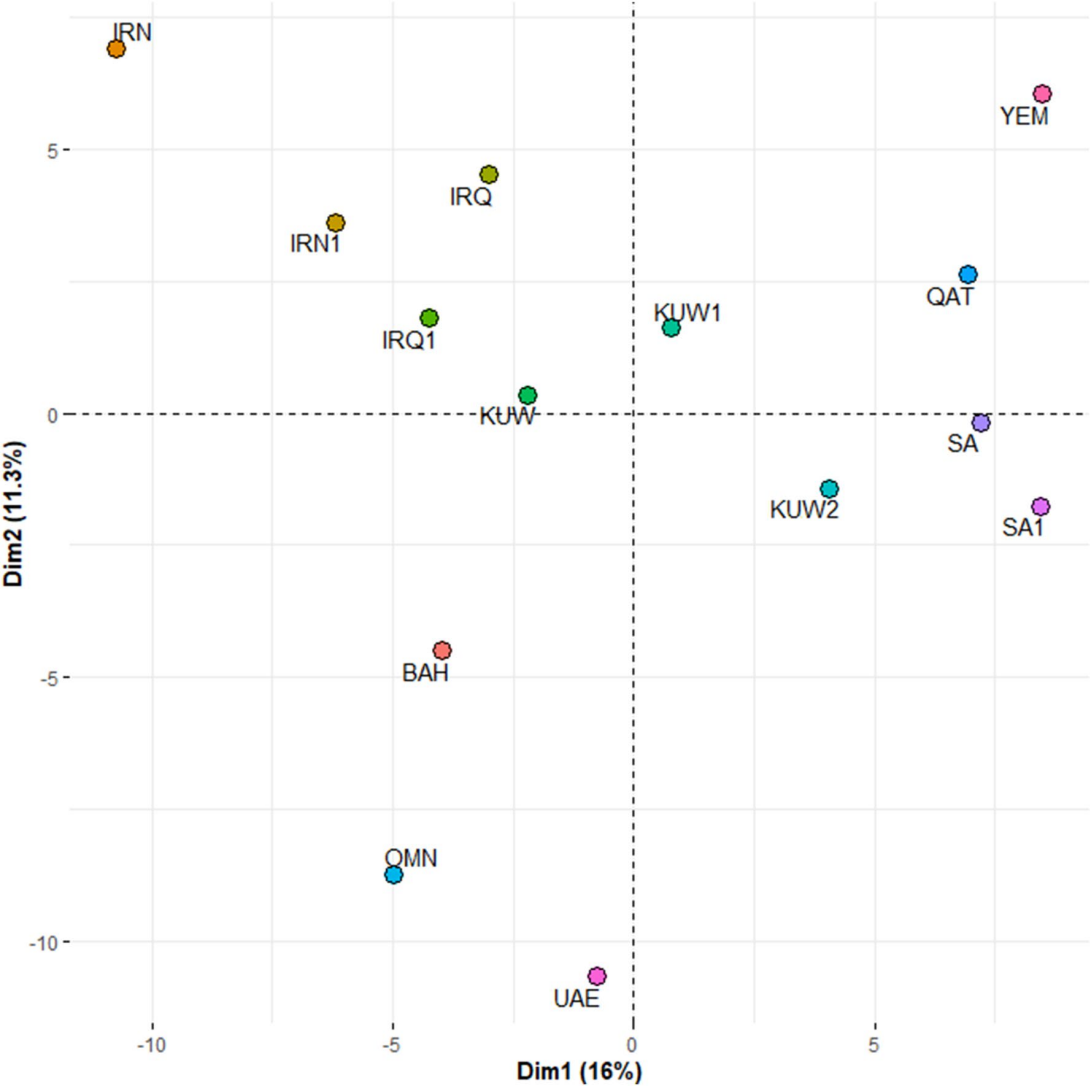
Principal component analysis of genetic distance based on allele frequencies of 13 autosomal PowerPlex Fusion 6C loci (CSF1PO, D13S317, D16S539, D18S51, D21S11, D3S1358, D5S818, D7S820, D8S1179, FGA, TH01, TPOX, vWA) shared between the dataset reported here (KUW), previously published Kuwaiti studies (KUW1 and KUW2), and studies of the populations of neighbouring countries Iraq (IRQ and IRQ1), Iran (IRN and IRN1), Saudi Arabia (SA and SA1), United Arab Emirates (UAE), Oman (OMN), Yemen (YEM), and Bahrain (BAH) (generated using *factoextra* package^34^ in R software^33^).

**Figure 4:**
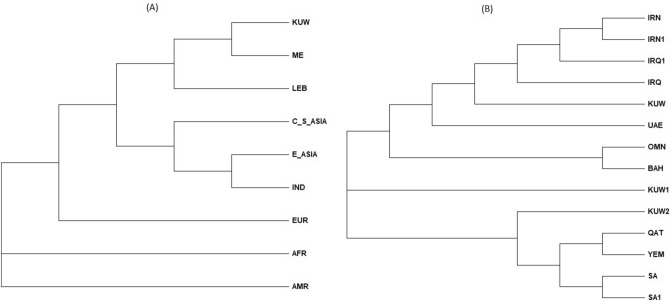
Neighbour-joining tree based on pairwise *D*_*A*_ values from allele frequencies of (**A**) the 23 autosomal PowerPlex Fusion 6C loci in Kuwait and populations from Africa (AFR), America (AMR), Central and South Asia (C_S_ASIA), the Middle East (ME), Europe (EUR), East Asia (E_ASIA), Lebanon (LEB) and India (IND; Madhya Pradesh), and (B) the 13 loci shared between the dataset reported here (KUW), previously published Kuwaiti studies (KUW1 and KUW2), and studies of the populations of neighbouring countries Iraq (IRQ and IRQ1), Iran (IRN and IRN1), Saudi Arabia (SA and SA1), United Arab Emirates (UAE), Oman (OMN), Yemen (YEM), and Bahrain (BAH). The nodes represent a common ancestor and the branching tips are descendants of that common ancestry (generated using *Mega X* software version 10.0.5^37^).

At the regional level, genetic distance was assessed based on the 13 loci shared between this study and studies examining other populations from the Arabian Peninsula. The resulting PCA plot (Fig. 3), and NJ tree (Fig. 4B) show that our Kuwaiti dataset broadly clustered with the previously published Kuwaiti data, and was genetically closer to the countries in the north of the region such as Iraq and Iran. In contrast, there was a higher level of genetic differentiation between Kuwait and Saudi Arabia, Yemen and Qatar, which were clustered in the upper-right part of the PCA plot, and Bahrain, Oman and UAE, which were clustered in the lower part of the plot.

In this study, 30% of individuals declared their origins as being from the north (Iraq and Iran), 39% from the south region (Saudi Arabia, Bedouin and Bahrain), and 24% had parents of different origin (admixed). Therefore, the closer genetic relationship of our samples to the northern region might be due to the presence of these individuals. There is no information available about the population of origin for the samples collected in the two previous Kuwaiti studies (KW1^3^ and KW2^2^). It is therefore not possible to determine whether sampling from different sub-populations could explain why, in contrast to our sample, these two Kuwaiti samples cluster more closely with the Saudi Arabian sample than the samples from Iran and Iraq. Overall, it can be seen that the allele frequencies of the 23 autosomal markers in the PowerPlex Fusion 6C kit can be successfully used to separate both geographically distant global populations and closely related populations on the basis of their genetic distance, making them a good choice for detecting genetic differentiation between populations.

## Conclusion

This study evaluated the forensic utility of the 23 autosomal STR loci included in the Promega PowerPlex Fusion 6C kit for the Kuwaiti population. Among these loci, D10S1248, D22S1045, D2S441 and SE33 are reported for the first time for Kuwait. The genetic data indicate that these 23 autosomal STRs are highly polymorphic in the Kuwaiti population and are of high value for human identification and paternity testing. *STRUCTURE* and PCA analysis show no signature of genetic structuring of the Kuwaiti population into subpopulations. Comparison of the Kuwaiti population to other global populations indicates that Kuwait clusters with other Middle Eastern populations, and shows a close relationship with Iran and Iraq, suggesting that they may share common ancestry.

## Supplementary Information

Supplementary Information

## References

[1] Alenizi M, Goodwin W, Ismael S, Hadi S (2008). STR data for the AmpFℓSTR® Identifiler® loci in Kuwaiti population. Leg. Med..

[2] Al-Enizi M (2013). Population genetic analyses of 15 STR loci from seven forensically-relevant populations residing in the state of Kuwait. Forensic Sci. Int. Genet..

[3] Al-enizi M (2014). Population data on 25 autosomal STRs for 500 unrelated Kuwaitis. Forensic Sci. Int. Genet..

[4] Hares DR (2015). Selection and implementation of expanded CODIS core loci in the United States. Forensic Sci. Int. Genet..

[5] Ensenberger MG (2016). Developmental validation of the PowerPlex® Fusion 6C System. Forensic Sci. Int. Genet..

[6] Excoffier L, Lischer HE (2010). Arlequin suite ver 3.5: A new series of programs to perform population genetics analyses under Linux and Windows. Mol. Ecol. Resour..

[7] Gouy A, Zieger M (2017). STRAF-A convenient online tool for STR data evaluation in forensic genetics. Forensic Sci. Int. Genet..

[8] Teebi AS (1994). Autosomal recessive disorders among Arabs: An overview from Kuwait. J. Med. Genet..

[9] Alsmadi O (2013). Genetic substructure of Kuwaiti population reveals migration history. PLoS One.

[10] Balding DJ (2005). Weight-of-Evidence for Forensic DNA Profiles.

[11] Pritchard JK, Stephens M, Donnelly P (2000). Inference of population structure using multilocus genotype data. Genetics.

[12] Falush D, Stephens M, Pritchard JK (2003). Inference of population structure using multilocus genotype data: Linked loci and correlated allele frequencies. Genetics.

[13] Hubisz MJ, Falush D, Stephens M, Pritchard JK (2009). Inferring weak population structure with the assistance of sample group information. Mol. Ecol. Resour..

[14] Triki-Fendri S (2010). Genetic structure of Kuwaiti population revealed by Y-STR diversity. Ann. Hum. Biol..

[15] Theyab JB, Al-Bustan S, Crawford MH (2012). The genetic structure of the Kuwaiti population: mtDNA Inter- and intra-population variation. Hum. Biol..

[16] John SE (2015). Kuwaiti population subgroup of nomadic Bedouin ancestry-Whole genome sequence and analysis. Genom. Data.

[17] Triki-Fendri S (2016). Genetic structure of the Kuwaiti population revealed by paternal lineages. Am. J. Human Biol..

[18] Evanno G, Regnaut S, Goudet J (2005). Detecting the number of clusters of individuals using the software STRUCTURE: A simulation study. Mol. Ecol..

[19] Kopelman NM, Mayzel J, Jakobsson M, Rosenberg NA, Mayrose I (2015). Clumpak: A program for identifying clustering modes and packaging population structure inferences across K. Mol. Ecol. Resour..

[20] Earl DA, von Holdt BM (2012). STRUCTURE HARVESTER: A website and program for visualizing STRUCTURE output and implementing the Evanno method. Conserv. Genet. Resour..

[21] Amigo J (2009). pop.STR—an online population frequency browser for established and new forensic STRs. Forensic Sci. Int. Genet. Suppl. Ser..

[22] Phillips C (2014). Global population variability in Qiagen Investigator HDplex STRs. Forensic Sci. Int. Genet..

[23] Phillips C (2011). Analysis of global variability in 15 established and 5 new European Standard Set (ESS) STRs using the CEPH human genome diversity panel. Forensic Sci. Int. Genet..

[24] Dixit S (2019). Forensic genetic analysis of population of Madhya Pradesh with PowerPlex Fusion 6C^TM^ Multiplex System. Int. J. Legal Med..

[25] Hedjazi A, Nikbakht A, Hosseini M, Hoseinzadeh A, Hosseini SM (2013). Allele frequencies for 15 autosomal STR loci in Fars province population, southwest of Iran. Legal Med. (Tokyo, Jpn.).

[26] Khubrani YM, Wetton JH, Jobling MA (2019). Analysis of 21 autosomal STRs in Saudi Arabia reveals population structure and the influence of consanguinity. Forensic Sci. Int. Genet..

[27] Alsafiah HM, Goodwin WH, Hadi S, Alshaikhi MA, Wepeba PP (2017). Population genetic data for 21 autosomal STR loci for the Saudi Arabian population using the GlobalFiler((R)) PCR amplification kit. Forensic Sci. Int. Genet..

[28] Perez-Miranda AM, Alfonso-Sanchez MA, Pena JA, Herrera RJ (2006). Qatari DNA variation at a crossroad of human migrations. Hum. Hered..

[29] Alshamali F, Alkhayat AQ, Budowle B, Watson ND (2005). STR population diversity in nine ethnic populations living in Dubai. Forensic Sci. Int..

[30] Jones RJ, Tayyare WA, Tay GK, Alsafar H, Goodwin WH (2017). Population data for 21 autosomal short tandem repeat markers in the Arabic population of the United Arab Emirates. Forensic Sci. Int. Genet..

[31] Al-Snan NR, Messaoudi S, Babu SR, Bakhiet M (2019). Population genetic data of the 21 autosomal STRs included in GlobalFiler kit of a population sample from the Kingdom of Bahrain. PLoS One.

[32] Farhan MM, Hadi S, Iyengar A, Goodwin W (2016). Population genetic data for 20 autosomal STR loci in an Iraqi Arab population: Application to the identification of human remains. Forensic Sci. Int. Genet..

[33] Team, R. C. *R: A Language and Environment for Statistical Computing*. https://www.R-project.org/ (2019).

[34] Mundt, A. K. a. F. *factoextra: Extract and Visualize the Results of Multivariate Data Analyses*. https://CRAN.R-project.org/package=factoextra (2017).

[35] Nei M, Tajima F, Tateno Y (1983). Accuracy of estimated phylogenetic trees from molecular data. II. Gene frequency data. J. Mol. Evol..

[36] Takezaki N, Nei M, Tamura K (2014). POPTREEW: Web version of POPTREE for constructing population trees from allele frequency data and computing some other quantities. Mol. Biol. Evol..

[37] Kumar S, Stecher G, Li M, Knyaz C, Tamura K (2018). MEGA X: Molecular evolutionary genetics analysis across computing platforms. Mol. Biol. Evol..

[38] Butler JM (2009). The single most polymorphic STR Locus: SE33 performance in US populations. Forensic Sci. Int. Genet. Suppl. Ser..

[39] Alghafri R (2015). Population data for SE33 locus in United Arab Emirates Arab population. Forensic Sci. Int. Genet. Suppl. Ser..

[40] Butler JM (2008). New resources for the forensic genetics community available on the NIST STRBase website. Forensic Sci. Int. Genet. Suppl. Ser..

[41] Borsuk LA, Gettings KB, Steffen CR, Kiesler KM, Vallone PM (2018). Sequence-based US population data for the SE33 locus. Electrophoresis.

