# Supplementary material for: Population genetics and forensic utility of 23 autosomal PowerPlex Fusion 6C STR loci in the Kuwaiti population
Source: Sci Rep. 2021 Jan 21;11:1865. doi: 10.1038/s41598-021-81425-y (PMC7820400; doi:10.1038/s41598-021-81425-y)
Supplement: Supplementary file 1 — Supplementary Information [file 41598_2021_81425_MOESM1_ESM.docx]

**Population genetics and forensic utility of 23 autosomal PowerPlex Fusion 6C STR loci in the Kuwaiti population**

Mahdi Haidar^1,2,*^, Fatimah A. Abbas^1,2^, Hussain Alsaleh^1,2^, Penelope R. Haddrill^1^

_1_Centre for Forensic Science, Department of Pure and Applied Chemistry, University of Strathclyde, Glasgow, Scotland, UK

_2_Kuwait Identification DNA Laboratory (KIDL), General Department of Criminal Evidence, Ministry of Interior, Kuwait

*Correspondence: Mr. Mahdi Haidar, Centre for Forensic Science, Department of Pure and Applied Chemistry, University of Strathclyde, 204 George Street, Glasgow, G1 1XW, UK.

Email: [mahdi.haidar@strath.ac.uk](mailto:mahdi.haidar@strath.ac.uk)

**Supplementary Figure 1.** Estimated log likelihood plots of *STRUCTURE* results. (A) Mean log likelihood (±SD) of the data at each of *K* populations (Ln P(X|K)), based on five repeat runs at each value of *K*. (B) Rate of change (Delta *K*) of the log likelihood between each value of *K* (generated using *STRUCTURE HARVESTER,* <http://taylor0.biology.ucla.edu/structureHarvester/>)^1^.


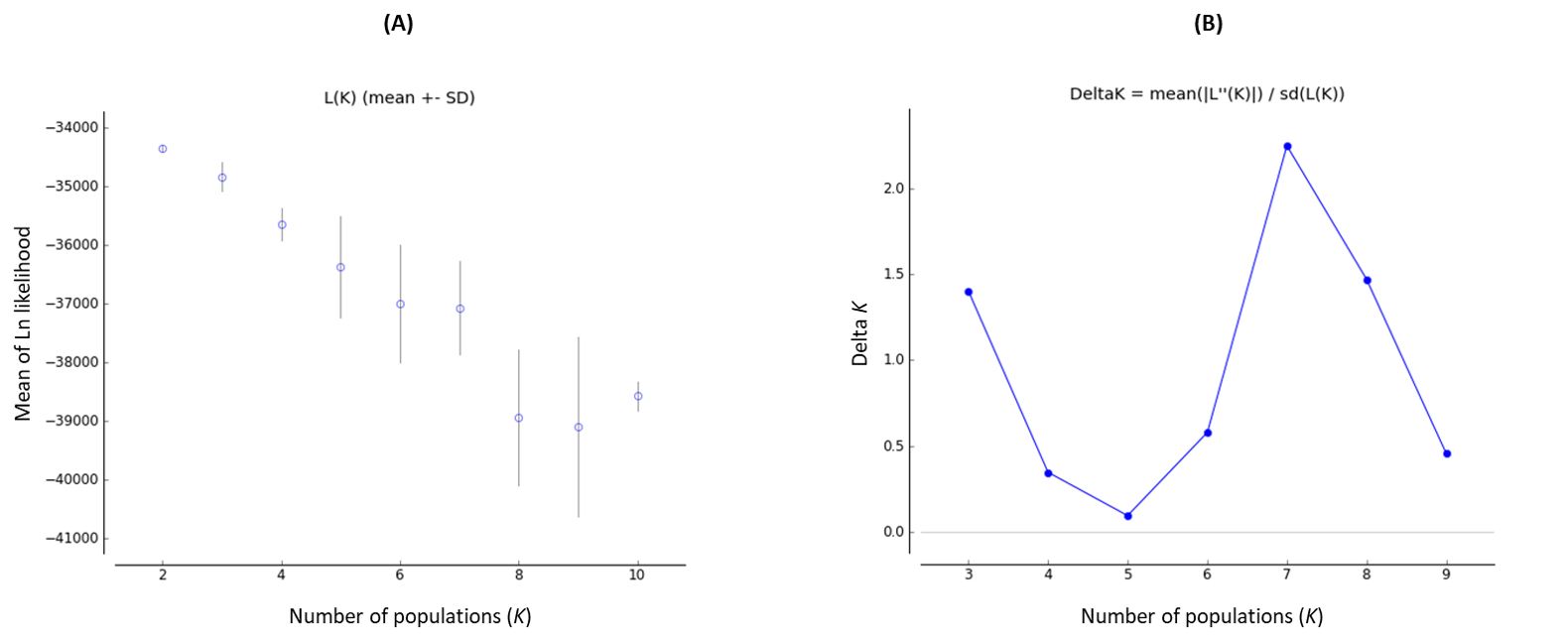


**Supplementary Figure 2.** Assessing population stratification in the Kuwaiti population using the model-based approach implemented in *STRUCTURE*. The number of colours in each bar represents the number of assumed populations (*K*) from 3 to 8, each vertical line represents an individual, and the amount of each colour in each line represents the proportion of that individual’s genetic ancestry that has been assigned to each of the *K* populations (generated using *CLUMPAK,* <http://clumpak.tau.ac.il/index.html>)^2^.


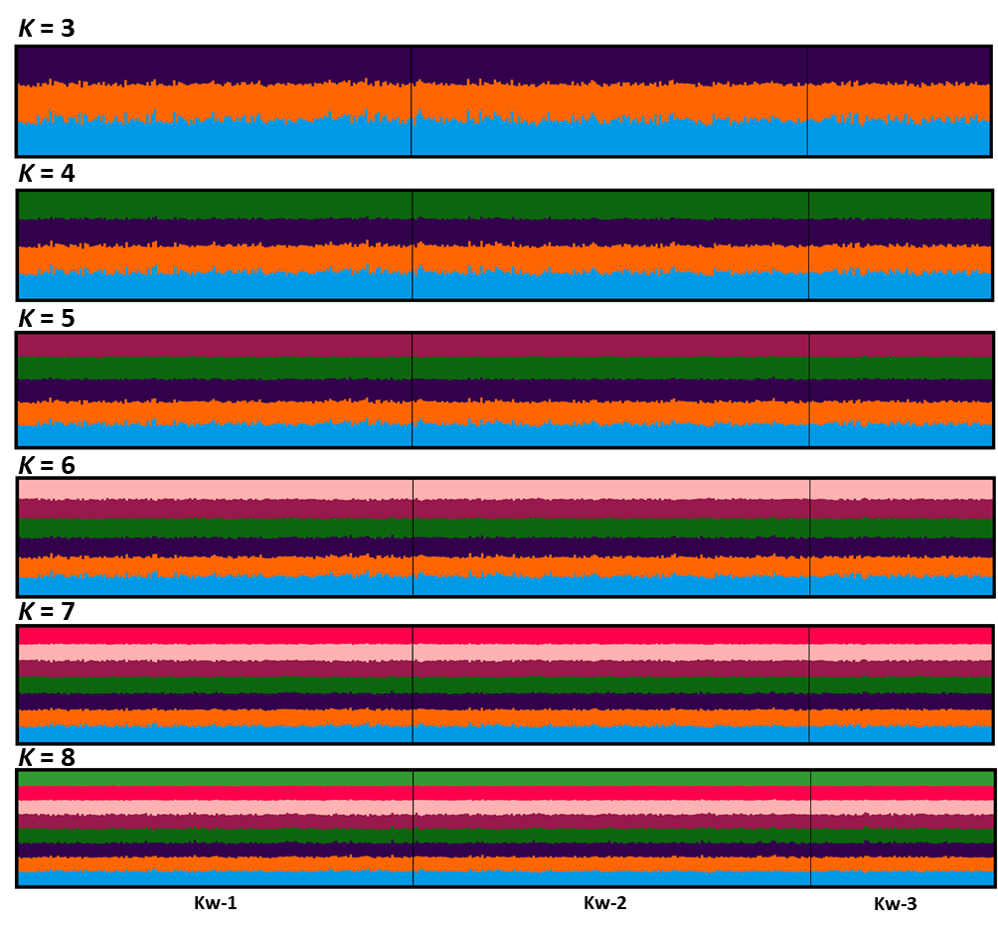


# **References**

1 Earl, D. A. & vonHoldt, B. M. STRUCTURE HARVESTER: a website and program for visualizing STRUCTURE output and implementing the Evanno method. *Conservation Genetics Resources* **4**, 359-361, doi:10.1007/s12686-011-9548-7 (2012).

2 Kopelman, N. M., Mayzel, J., Jakobsson, M., Rosenberg, N. A. & Mayrose, I. Clumpak: a program for identifying clustering modes and packaging population structure inferences across K. *Molecular ecology resources* **15**, 1179-1191, doi:10.1111/1755-0998.12387 (2015).
